# Appetitive and aversive motivation in depression: The temporal dynamics of task-elicited asymmetries in alpha oscillations

**DOI:** 10.1038/s41598-019-53639-8

**Published:** 2019-11-20

**Authors:** Simone Messerotti Benvenuti, Giulia Buodo, Rocco Mennella, Elisa Dal Bò, Daniela Palomba

**Affiliations:** 1 Department of General Psychology, University of Padua, Padua, Italy; 2 Laboratoire de neurosciences cognitives, Département d’études cognitives, École normale supérieure, INSERM, PSL Research University, 75005 Paris, France

**Keywords:** Emotion, Human behaviour, Depression

## Abstract

The capability model of alpha asymmetries posits that state emotional manipulations are a more powerful detector of depression-related motivational deficits than alpha activity at rest. The present study used a time-frequency approach to investigate the temporal dynamics of event-related changes in alpha power during passive viewing of emotional pictures in individuals with dysphoria (*n* = 23) and in individuals without dysphoria (*n* = 24). In the whole group, the processing of pleasant and unpleasant compared to neutral pictures was associated with a decrease in event-related alpha power (i.e., alpha desynchronization) at centro-parietal and parietal scalp sites in the 538–1400 ms post-stimulus. The group with dysphoria revealed a smaller alpha desynchronization than the group without dysphoria in response to pleasant, but not neutral and unpleasant, stimuli at frontal, fronto-central and centro-parietal sites. Interestingly, at central and centro-parietal scalp sites, the difference between groups in response to pleasant stimuli was lateralized to the right hemisphere, whereas no clear lateralization was observed at frontal and fronto-central scalp sites. These findings suggest that decreased cortical activity (i.e., reduced alpha desynchronization) in a network involving bilateral frontal and right-lateralized parietal regions may provide a specific measure of deficits in approach-related motivation in depression.

## Introduction

Depression is characterized by excessive and persistent negative mood, and/or by anhedonia and loss of pleasure in daily activities^1^. It has been proposed that both emergence and maintenance of depressive symptoms are accounted for by a preferential processing bias for mood-congruent (i.e., negative) information (for a review see Clark & Beck^2^). Such bias is supposed to produce a dominance of negative or threat-related thoughts, images and interpretations, in line with the idea that negative mood potentiates like-valenced or matching emotions^3^, known as the negative potentiation hypothesis.

Despite the empirical support to this conceptualization^4–6^, recent evidence suggests, on the contrary, that depressive symptoms emerge mostly as a consequence of reduced emotional response to positively valenced and rewarding stimuli^7,8^, possibly indicating a dysregulation of the approach-related motivational system in the brain^9,10^. Importantly, reduced approach-related motivation constitutes an important risk factor for clinical depression^11^ and – despite its scarce consideration in clinical practice – it may account for core symptoms such as anhedonia, apathy and loss of interests (for a review see Admon & Pizzagalli^12^). This model, known as the positive attenuation hypothesis, has been recently extended by a third alternative that postulates that depression is characterized by blunted reactivity to all emotional stimuli regardless of their valence, the so-called emotional context insensitivity (ECI)^13,14^. Specifically, the ECI model holds that individuals with depression exhibit reduced reactivity in response to both pleasant and, in contrast with the negative potentiation hypothesis, unpleasant stimuli, as a result of underactivation of the appetitive and the defensive motivational systems, respectively.

Reduced approach-related motivation in subclinical^15,16^ and clinical depression^17,18^, as well as in euthymic participants with a history of depression^19,20^, has been associated with hypoactivity of the left frontal lobe compared to the right. These findings support a renowned conceptual model which proposed that left frontal activity subtends the propensity to approach or engage a stimulus, whether reduced left vs. right frontal activity indicates a reduction in approach behaviors and increased withdrawal motivation^21–24^. Unbalanced cortical activity between the frontal areas of the two hemispheres is typically measured as asymmetry in the alpha band, a brain rhythm associated with cortical inhibition^25^. Accordingly, a recent EEG study confirmed that alpha asymmetry measured from the scalp correlated with asymmetry in the activation of lateral mid-frontal regions of the brain, and that participants with a history of depressive episodes were characterized by less left relative to right cortical activity in these regions^26^.

So far, the vast majority of studies on frontal alpha asymmetry in depression have investigated reduced approach-related motivation at rest. This is consistent with a *dispositional* model of motivation and affective style, which proposes that individuals have a trait-like tendency to respond with either approach or withdrawal, irrespective of the specific demands of the situation^23^. Nonetheless, inconsistent results have emerged, raising criticisms about the effective value of resting frontal alpha asymmetry as a potential biomarker for depression (for a recent meta-analysis see van der Vinne *et al*.^27^). Following these concerns, a *capability* model has been proposed, which states that individuals differ in their emotion regulatory abilities in situations with specific emotional charges^28^. In other words, reduced approach-related motivation in depression is thought to be more evident in response to emotional stimuli than at rest, since the emotional demands of the context highlight the motivational deficit, and reduce undesirable variance associated with resting states^25,28,29^.

To date, only few studies have started investigating alpha asymmetry in depression in emotion- or reward-related tasks^9,29–31^. Moreover, two methodological shortcomings ought to be mentioned. First, EEG activity has typically been averaged over several seconds, providing no information regarding the time course of the response to emotional stimuli. This is surprising considering that emotional responding and regulation occur within a few hundred of millisecond, and considering that alpha asymmetry has been reported to burst transiently also at rest^32^. Furthermore, recent studies have analyzed alpha activity only at anterior scalp sites, even if asymmetry in the alpha band in depression has been reported also at posterior scalp sites, despite some exception^33^. In particular, depression (current or remitted) and familiarity for depression are characterized by a right temporo-parietal dysfunction, as indicated by increased right relative to left parietal alpha activity^19,33,34^. Decreased right parietal activity is thought to reflect reduced arousal and impaired processing of emotional stimuli^33,35–37^, and may thus concur to emotional dysregulation in depression.

A time-frequency analysis of the EEG response to emotional stimuli allows to overcome most of these limitations. Importantly, frontal alpha response to emotional stimuli can be evaluated over time, overcoming the “static picture” provided by conventional fast Fourier transform (FFT) spectral analysis based on averaging procedures. Interestingly, this method has been proven successful in detecting transient motivational responses to phobic pictures in specific phobia^38^, as indicated by alpha power at frontal sites. More specifically, the time-frequency approach allows to assess how depression-related motivational disposition affects alpha power in response to emotional stimuli with an excellent temporal resolution (in the millisecond range).

The goal of the present study was to investigate motivational deficits in depression through the analysis of the time-frequency changes in response to emotional stimuli from the International Affective Picture System (IAPS) library^39^, according to the capability model of alpha asymmetries. So far, affective pictures processing in a passive viewing task has been assessed mostly with regard to distinct components of the event-related potentials (ERPs). In particular, compared with low-arousing neutral stimuli, high-arousing pleasant and unpleasant stimuli typically elicit larger P3 and late positive potential (LPP) amplitudes over the centro-parietal regions in the 300–700 ms time window, which are thought to reflect attentional processing of emotional stimuli^40–42^. In the present study, pictures (pleasant, neutral and unpleasant) were selected to elicit robust P3/LPP complex, serving as an experimental manipulation check. The group with dysphoria was expected to show a smaller decrease in event-related alpha power (i.e., a reduced alpha desynchronization) in the left frontal and the right posterior regions in response to pleasant (but not neutral and unpleasant) pictures compared to controls, as a correlate of reduced approach-related motivation. Given that the negative potentiation model and the ECI model make two opposite predictions with respect to depression-related emotional reactivity in response to negative stimuli, no a priori hypothesis was formulated regarding the direction of changes in event-related alpha power in response to unpleasant pictures.

## Methods

### Participants

The method used to recruit participants was based on that described in a previous study by Messerotti Benvenuti *et al*.^10^ Specifically, in order to preliminary identify participants with dysphoria, 197 undergraduate students from the University of Padua completed an online version of the Beck Depression Inventory-II (BDI-II^43^; Italian version by Ghisi *et al*.^44^). The BDI-II is a reliable and valid self-report questionnaire that evaluates the severity of symptoms of depression in the past two weeks. Answers are given on a four-point (0–3) Likert scale and scores range from 0 to 63, with the higher scores indicating more severe depressive symptoms. In the Italian version, a score of 12 has been reported as the optimal cut-off score to discriminate individuals with and without depressive symptoms^44^. Participants scoring equal to or greater than 12 on the online version of BDI-II (*n* = 77) were invited to participate in the study and were administered a paper-and-pencil version of the BDI-II and the mood episode module (module A) of the Structured Clinical Interview for the DSM-IV Axis I (SCID-I^45^; Italian version by Mazzi *et al*.^46^) approximately one week after the initial screening. The module A of the SCID-I was administered to confirm the presence of dysphoria and to exclude individuals with major depression, dysthymia or bipolar disorder. The module A of the SCID-I was administered by a trained psychologist who had previous experience with administering structured clinical interviews. Twenty-three participants [22 females and 1 male; age, mean (*M*) = 21.9, standard deviation (*SD*) = 2.2; BDI-II score, *M* = 17.3, *SD* = 4.4], who scored equal to or greater than 12 on both versions of the BDI-II and had at least two current depressive symptoms, at least two weeks in duration, without meeting the diagnostic criteria for major depression, dysthymia or bipolar disorder, were assigned to the group with dysphoria. In order to ensure separation between groups with and without dysphoria, we selected 24 individuals without dysphoria [23 female and 1 male; age, *M* = 22.0, *SD* = 1.9; BDI-II score, *M* = 2.6, *SD* = 1.9] with an online BDI-II score ≤8 (corresponding to the 45° percentile) and confirmed in the subsequent administration of the paper-and-pencil version of the BDI-II. Participants who scored between 9 and 11 either on the online or the paper-and-pencil BDI-II, or had at least one depressive symptom as evaluated by the SCID-I interview were excluded from the present study.

All the participants enrolled in the present study met the following inclusion criteria as assessed by an ad-hoc interview: 1) being medically healthy, and 2) being free of psychotropic medications. With respect to demographic variables, the two groups (with dysphoria, without dysphoria) did not differ in terms of gender (Fisher’s exact test, *p* = 0.99) and age, F_(1,45)_ = 0.05, *p* = 0.83, *η*^2^_*p*_ = 0.00. The group with dysphoria showed significantly higher BDI-II scores than the group without dysphoria, F_(1,45)_ = 226.69, *p* < 0.001, *η*^2^_*p*_ = 0.83.

### Ethics statement and informed consent

The present study was conducted with the adequate understanding and written consent of the participants in accordance with the Declaration of Helsinki. The study was approved by the local Ethics Committee, University of Padua (prot. No. 2101), and written informed consent was obtained from each participant enrolled in the study.

### Stimuli and procedure

Participants were presented seventy-two pictures selected from the IAPS^39^, divided into three categories: 24 pleasant (e.g., erotic scenes, sports), 24 neutral (e.g., neutral faces, household objects), and 24 unpleasant (e.g., attacking humans and animals). The pictures were selected on the basis of their standardized ratings of affective arousal and valence. The mean (*SD*) normative valence ratings were 7.0 (0.5), 4.9 (0.3) and 2.9 (0.7) for pleasant, neutral and unpleasant pictures, respectively. The mean (*SD*) normative arousal ratings were 6.5 (0.4), 2.9 (0.7) and 6.5 (0.5) for pleasant, neutral and unpleasant pictures, respectively. Pleasant and unpleasant stimuli were matched for arousal (*p* = 0.92). The IAPS picture numbers were 1050, 1114, 1120, 1300, 1302, 1930, 1932, 3500, 4611, 4647, 4651, 4652, 4660, 4664, 4670, 4680, 4683, 4690, 4695, 4810, 6200, 6210, 6230, 6242, 6243, 6244, 6250, 6260, 6312, 6313, 6370, 6510, 6540, 6550, 6560, 7000, 7002, 7004, 7009, 7010, 7020, 7035, 7036, 7041, 7050, 7056, 7059, 7130, 7175, 7224, 7233, 7242, 7491, 7500, 7547, 7560, 7595, 7700, 7950, 8030, 8031, 8034, 8080, 8161, 8180, 8185, 8186, 8200, 8370, 8400, 8490, 9425.

Pictures were presented for 6,000 ms each in a semi-randomized sequence (i.e., no more than one stimulus in the same emotional condition had to be shown consecutively). Each picture was preceded by a 3,000-ms gray interval with a white fixation-cross placed centrally on the screen. In order to ensure that participants processed each picture’s content, they were required to look at the central fixation-cross and keep their gaze on the center of the screen. An acoustic startle probe was presented at one of four intervals (i.e., 300, 1500, 3500 or 4500 ms after picture onset) on each trial, thus providing 6 data points for each time condition within each emotional category. The data analysis did not include trials on which a startle probe was delivered at 300 ms after pictures onset. Therefore, six stimuli for each emotional condition were excluded from the analysis. The startle reflex (and heart rate) data are not presented here. The inter-stimulus interval was randomly varied between 6,000 and 8,000 ms. The task was presented by a Pentium IV computer on a 19-in. computer screen, using E-prime 2.0 presentation software (Psychology Software Tools, Pittsburgh, PA, USA).

According to the procedure reported in a previous study by Messerotti Benvenuti *et al*.^10^, upon arrival at the laboratory, the participants were first administered a paper-and-pencil version of the BDI-II and the mood episode module (module A) of the SCID-I interview. Then, participants were seated 100 cm away from the computer monitor, in a dimly lit, sound-attenuated room. After the sensors were attached, six practice trials including two pleasant, two neutral, and two unpleasant pictures were provided. Then, each participant performed the emotional passive viewing task.

**Figure 1:**
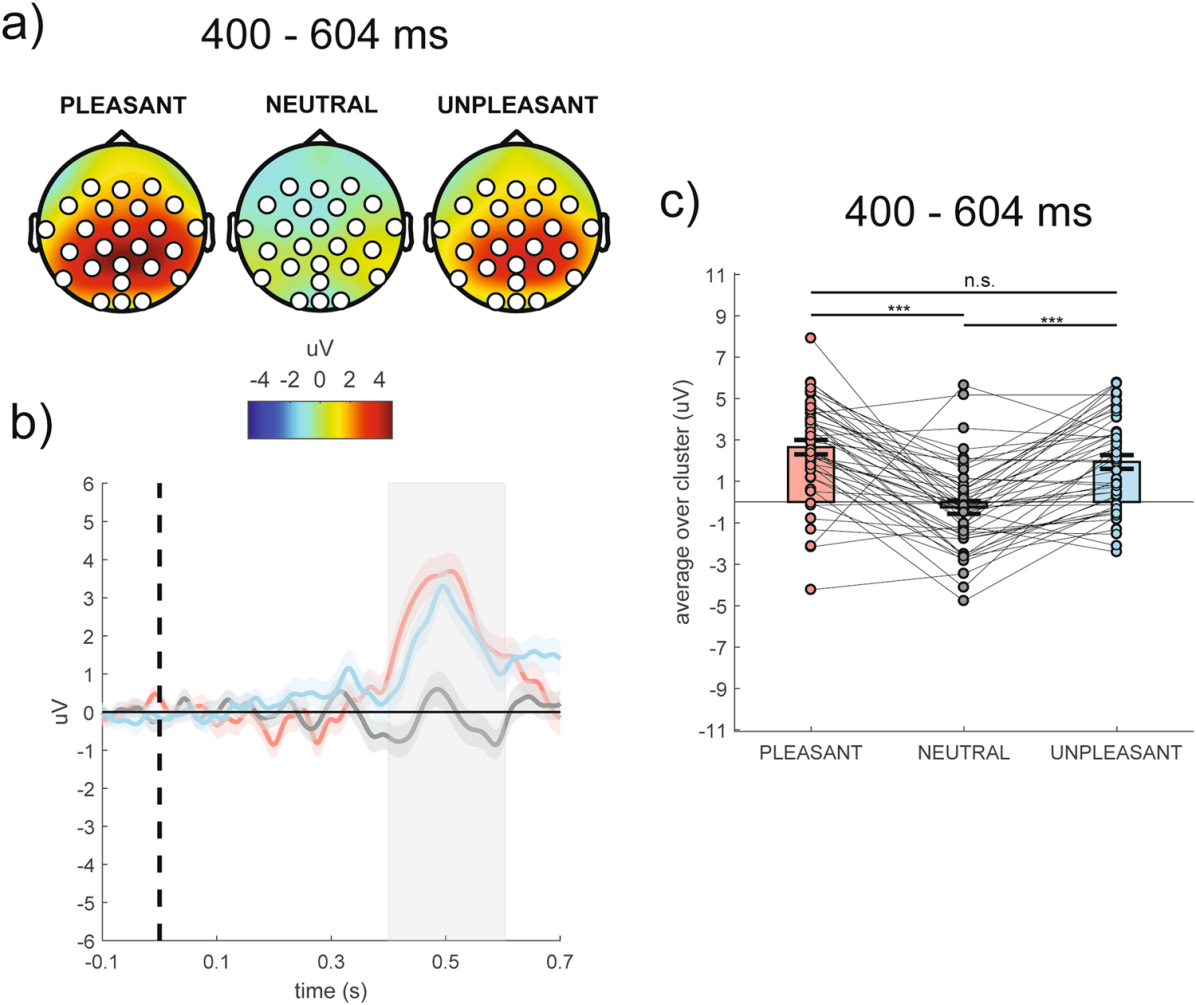
(Panel a) Topography of the mean ERP amplitude (μV) averaged over the significant time points (400–604 ms time window) for pleasant, neutral and unpleasant conditions. (Panel b) Time course of grand-average ERP waveforms averaged over the significant electrodes for pleasant (red line), neutral (grey line) and unpleasant (light blue line) conditions. Shaded areas represent ± standard error of the mean (SEM); the colored frame represents the significant time window (400–604 ms). (Panel c) Mean ERP amplitude of each participant averaged over the significant electrodes and time points for pleasant, neutral and unpleasant conditions. Each circle represents one participant; colored frames represent the mean ERP amplitude across all participants and the solid black lines represent ± SEM. ***p < 0.001.

At the end of the passive viewing task, 36 pictures (12 for each emotional category) were presented again in a randomized sequence, and ratings of emotional valence and arousal were obtained using a two computerized 9-point Self-Assessment Manikin (SAM) scales^47^. The SAM uses manikin figures for both valence and arousal dimensions. On the valence dimension, the SAM figures range from a frowning-unhappy figure (1, very unpleasant) to a smiling-happy figure (9, very pleasant). On the arousal dimension, the SAM figures range from a static-eyes-closed figure (1, very calm) to an active-wide-eyed figure (9, very aroused). Following completion of the self-evaluation of emotional valence and arousal, the participants were fully debriefed. The entire procedure lasted approximately 90 min.

### Electroencephalographic recordings

The EEG was recorded from 32 scalp sites using an elastic cap with tin electrodes (Waveguard EEG cap, ANT Neuro, Enschede, Netherlands). The EEG sites were FP1, FPz, FP2, F7, F3, Fz, F4, F8, FC5, FC1, FC2, FC6, T7, C3, Cz, C4, T8, CP5, CP1, CP2, CP6, P7, P3, Pz, P4, P8, POz, O1, Oz, O2, A1 (left mastoid) and A2 (right mastoid), all referenced online to CPz. To control for eye movements and eye blinks, vertical and horizontal electrooculograms (EOGs) were recorded using bipolar montages. Electrode pairs were placed at the supra- and suborbital right eye and at the external canthi of the eyes. Electrode impedance was kept below 10 kΩ. The EEG and EOG signals were amplified with eego amplifier (ANT Neuro, Enschede, Netherlands), bandpass filtered (0.3–40 Hz), and digitized at 1000 Hz.

### Data preprocessing

The EEG signal was downsampled to 500 Hz and re-referenced offline to a linked mastoids montage. The EEG was filtered offline with a low-pass filter at 30 Hz and manually corrected for blink artifacts using independent component analysis (ICA) as implemented in EEGLAB^48^. Further processing was conducted in Brainstorm^49^. The EEG was then segmented into 4,000 epochs, from 2,000 ms before to 2,000 ms after stimulus onset, in order to prevent boundary effects. Each epoch was baseline-corrected by subtracting the mean pre-stimulus voltage between −252 ms and −52 ms. Then, segments containing residual artifacts exceeding ±70 μV (peak-to-peak) were excluded. The artifact rejection led to an average (*SD*) acceptance for the ERP and for the time-frequency analyses of 17.0 (1.2) pleasant trials, 17.0 (1.1) neutral trials and 17.1 (1.0) unpleasant trials in the group with dysphoria, and of 16.6 (1.1) pleasant trials, 16.8 (1.2) neutral trials and 17.0 (0.9) unpleasant trials in the group without dysphoria. No significant differences between groups or among emotional conditions in the average acceptance of pleasant, neutral and unpleasant trials were noted (all *p*s > 0.25).

### Event-related potentials (ERPs)

ERPs were calculated by averaging EEG epochs in the time domain separately for each participant and emotional condition.

### Time-frequency analysis

With respect to the time-frequency analysis, Morlet wavelet transformation on individual trials was applied for each 1 Hz frequency bin between 1 and 20 Hz, using a mother wavelet at 1 Hz with 2-s time resolution (as calculated by the full width at half maximum; FWHM). Time-frequency decompositions were then averaged for each subject and emotional condition, and the event-related spectral perturbation (ERSP) was computed as the change in power expressed in decibels (dB) relative to the baseline (−500 to −52 ms) in each frequency bin at each time point. Then, data were grand averaged across participants with dysphoria and across participants without dysphoria for each emotional condition.

### Statistical analysis

#### Self-report data

Separate mixed analyses of variance (ANOVAs) with Group (with dysphoria, without dysphoria) as a between-subjects factor, and Category (pleasant, neutral, unpleasant) as a within-subjects factor, were conducted on self-reported valence and arousal. The corrected *p*-values for effects involving within-subjects variables with more than two levels are reported together with the Greenhouse-Geisser epsilon (ε) and the uncorrected degrees of freedom. Significant main effects and/or interactions (*p* < 0.05) were followed by Tukey HSD post-hoc tests in order to correct for multiple comparisons. Cohen’s d (absolute value) for relevant comparisons was calculated as a measure of the effect size. All effect sizes, corrected for the sample bias^50^, are reported with 95% confidence intervals (CIs) and were considered significant if the CIs did not overlap zero.

#### EEG data: general method

In order to perform statistical analysis on EEG data, a cluster-based approach has been conducted to control over the type I error rate arising from multiple comparisons across electrodes and time points^51^. Statistical tests were run across electrodes and time points; the resulting values were thresholded and the differences among emotional conditions or groups were shuffled pseudo-randomly 2000 times. The maximal cluster-level statistics (i.e., the sum of values across contiguously significant electrodes and time points at the threshold level) were extracted for each shuffle to compute a ‘null’ distribution of effect sizes. For each significant cluster in the original (non-shuffled) data, it was computed the proportion of clusters in the null distribution whose statistics exceeded the one obtained for the cluster in question, corresponding to its cluster-corrected *p*-value. Clusters with a *p*_corr_ < 0.05 were considered statistically significant.

ERP data: Repeated measures ANOVAs over all electrodes and time-points in the −100 to 700 ms interval were employed to test differences in ERP amplitudes among emotional conditions (Category: pleasant, neutral, unpleasant), with the group variable collapsed. An initial conservative alpha of 0.001 was employed to threshold the matrices due to the expected large effect of emotional category on P3/LPP complex, in order to highlight the electrodes and time points where the difference was more prominent (note that this value does not affect the false alarm rate of the statistical test at the cluster-level^51^).

When the time window was identified, a second cluster-based analysis was run to test the differences between groups within each emotion category. In this analysis, a two-tailed unpaired t-test on the ERP amplitude averaged over the significant time window was conducted across electrodes for each emotional condition.

Time-frequency data: A similar cluster-based analysis was conducted on event-related alpha power (8–13 Hz), with a −100–1400 ms time window and a *p*_thresh_ = 0.05. Then, in order to perform analysis at the group level, the same cluster-based approach (statistic = one-tailed unpaired t-test) on the event-related alpha power averaged over the significant time points was conducted across electrodes for each emotional category. One-tailed t-test was used based on an a priori hypothesis about the direction of the difference between groups in event-related alpha power in response to pleasant stimuli.

In order to control for the specificity of the effects on alpha, the same statistical approach was conducted on event-related changes in delta (1–3 Hz), theta (4–7 Hz), and beta (14–20 Hz) power, using a two-tailed unpaired t-test.

## Results

### Self-report data

The mixed ANOVA on valence ratings yielded a significant main effect for Category, F_(2,90)_ = 280.76, *p* < 0.001, *ε* = 0.85, *η*_*p*_^2^ = 0.86. Unpleasant pictures were evaluated as significantly more unpleasant than neutral (*p* < 0.001; d = 3.36, 95% CI = 2.73, 3.99) and pleasant (*p* < 0.001; d = 4.11, 95% CI = 3.40, 4.82) pictures. Pleasant stimuli were rated as significantly more pleasant than neutral ones (*p* < 0.001; d = 1.49, 95% CI = 1.04, 1.95). No significant main effect for Group or Group × Category interaction was found (all *p*s > 0.11). Similarly, the ANOVA on arousal ratings revealed a significant main effect for Category F_(2,90)_ = 114.89, *p* < 0.001, *ε* = 0.83, *η*_*p*_^2^ = 0.72. Specifically, arousal ratings were higher for both pleasant and unpleasant pictures compared to neutral ones (pleasant vs. neutral: *p* < 0.001; d = 1.46, 95% CI = 1.01, 1.92; unpleasant vs. neutral: *p* < 0.001; d = 1.96, 95% CI = 1.47, 2.46). Unpleasant pictures were rated as more arousing than pleasant stimuli (*p* < 0.001; d = 0.54, 95% CI = 0.12, 0.95). However, it is important to note that this effect was driven by participants with dysphoria (d = 0.84, 95% CI = 0.24, 1.44) instead of controls, in which the effect size of the difference between the pleasant and unpleasant stimuli was small (d = 0.31, 95% CI = −0.26, 0.88). No significant main effect for Group or Group × Category interaction was found (all *p*s > 0.18). The descriptive statistics of self-report measures are reported in Table 1.

**Table 1:** Ratings of each self-report measure in the group with dysphoria and in the group without dysphoria.

Self-report measure	Group with dysphoria (*n* = 23)			Group without dysphoria (*n* = 24)		
	Pleasant	Neutral	Unpleasant	Pleasant	Neutral	Unpleasant
Valence	6.3 (1.0)	5.3 (0.5)	2.7 (1.2)	6.7 (0.8)	5.3 (0.7)	2.4 (0.8)
Arousal	5.1 (1.4)	2.8 (1.7)	6.4 (1.5)	4.9 (1.8)	2.6 (1.5)	5.5 (1.8)

*Note*. Data are *M* (*SD*).

### ERP data

#### Differences among emotional categories

The cluster-based analysis on ERP data showed a significant positive fronto-centro-parieto-occipital cluster (cluster F-value_max_ = 63009.06, *p*_corr_ < 0.001, time window = 400–604 ms, electrodes = F3, Fz, F4, FC5, FC1, FC2, FC6, T7, C3, Cz, C4, T8, CP5, CP1, CP2, CP6, P7, P3, PZ, P4, P8, POz, O1, Oz, O2), as shown in Fig. 1 (panel a). Specifically, the whole group revealed a significantly larger P3/LPP complex in response to pleasant and unpleasant stimuli than neutral ones (all *p*s < 0.001; Fig. 1, panels b,c), especially at central, centro-parietal and parietal scalp sites (Fig. 1, panel a).

#### Differences between groups for each emotional category

Unpaired t-test conducted on the P3/LPP amplitude averaged over the 400–604 ms time window, where the effect of emotion emerged in the previous analysis, did not reveal any significant cluster for the difference between the groups within each emotional condition.

### Time-frequency data

#### Differences among emotional categories in event-related alpha power

**Figure 2:**
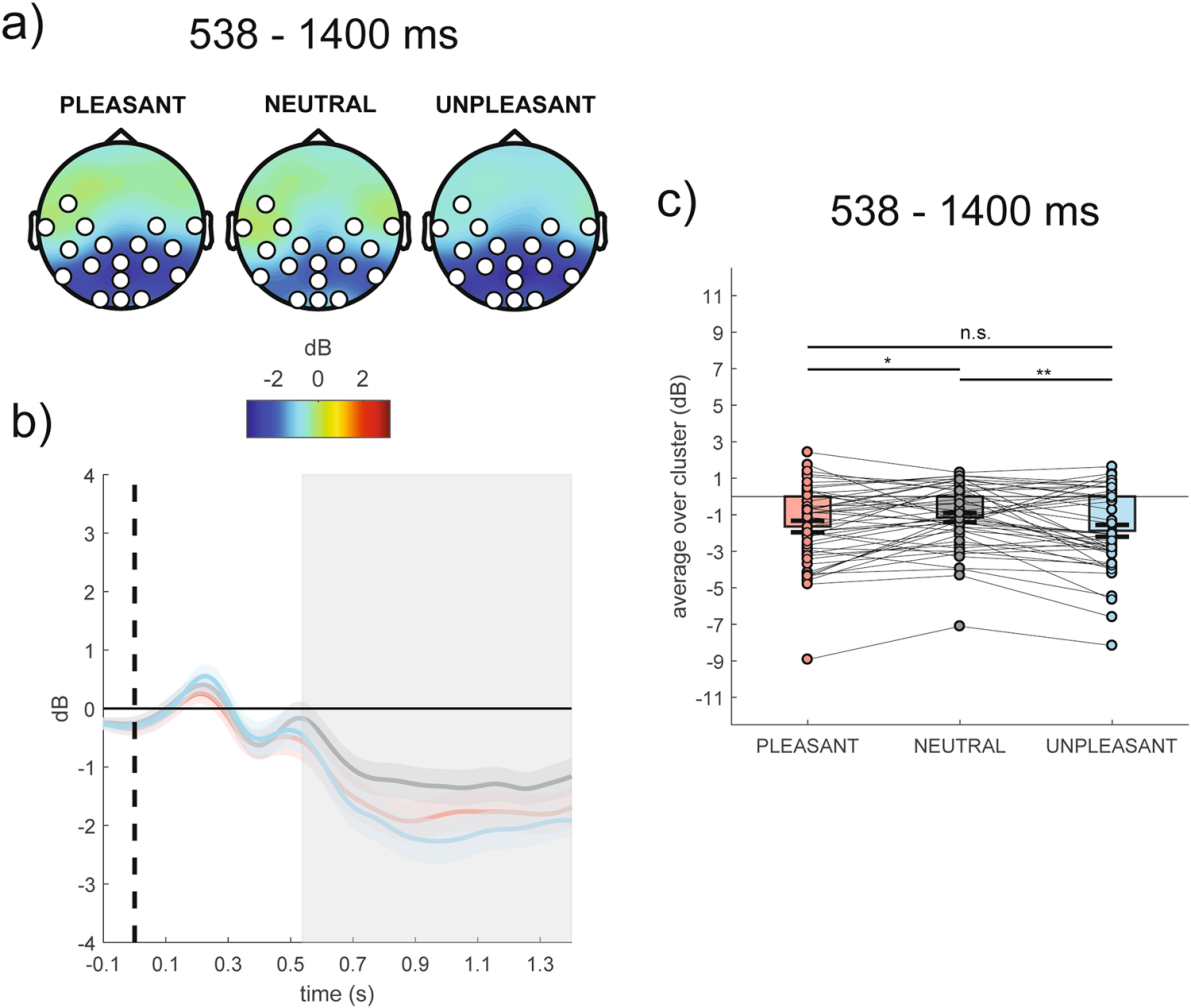
(Panel a) Topography of the mean event-related alpha power (dB) averaged over the significant time points (538–1400 ms time window) for pleasant, neutral and unpleasant conditions. (Panel b) Time course of grand-average event-related alpha power averaged over the significant electrodes for pleasant (red line), neutral (grey line) and unpleasant (light blue line) conditions. Shaded areas represent ± standard error of the mean (SEM); the colored frame represents the significant time window (538–1400 ms). (Panel c) Mean event-related alpha power of each participant averaged over the significant electrodes and time points for pleasant, neutral and unpleasant conditions. Each circle represents one participant; colored frames represent the mean event-related alpha power across all participants and the solid black lines represent ± SEM. *p < 0.05; **p < 0.01.

In the whole group, the cluster-based analysis on event-related alpha power revealed a significant positive centro-parieto-occipital cluster (cluster F-value_max_ = 37097.61, *p*_corr_ = 0.01, time window = 538–1400 ms, electrodes = FC5, T7, C3, C4, T8, CP5, CP1, CP2, CP6, P7, P3, Pz, P4, P8, POz, O1, Oz, O2), as shown in Fig. 2 (panel a). In particular, reduced event-related alpha power was evident in response to pleasant and unpleasant stimuli compared to neutral ones (all *p*s < 0.05), as shown in Fig. 2 (panels b,c).

#### Differences between groups in event-related alpha power for each emotional category

**Figure 3:**
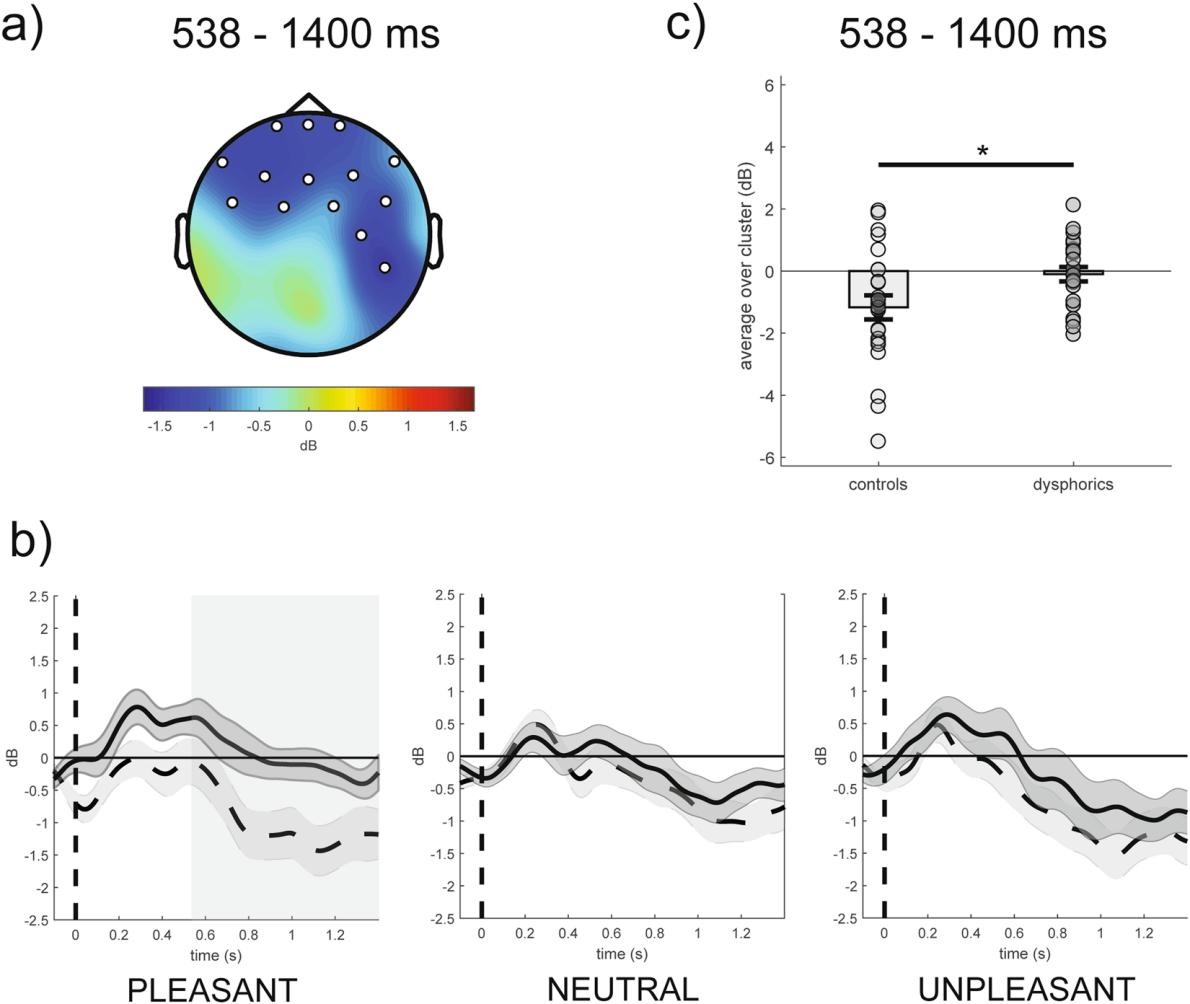
(Panel a) Topography of the mean difference between groups in event-related alpha power (dB; group without dysphoria minus group with dysphoria) averaged over the significant time points (538–1400 ms time window) for the pleasant condition. (Panel b) Time course of grand-average event-related alpha power averaged over the significant electrodes for pleasant, neutral and unpleasant conditions in the group with dysphoria (solid line) and in the group without dysphoria (dashed line). Shaded areas represent ± standard error of the mean (SEM); the colored frame represents the significant time window (538–1400 ms). (Panel c) Mean event-related alpha power of each participant in the group with dysphoria and in the group without dysphoria (i.e., controls) averaged over the significant electrodes and time points for the pleasant condition. Each circle represents one participant; the frames represent the mean event-related alpha power across all participants in the group with dysphoria and in the group without dysphoria and the solid black lines represent ± SEM. *p < 0.05.

With respect to the differences between groups for each emotional condition in the 538–1400 ms time window, the cluster-based analysis on event-related alpha power showed a significant negative fronto-centro-parietal cluster in the pleasant condition (cluster t-value_max_ = −29.24, *p*_corr_ = 0.02, electrodes = FP1, FPz, FP2, F7, F3, Fz, F4, F8, FC5, FC1, FC2, FC6, C4, CP6), as shown in Fig. 3 (panel a). Specifically, the group without dysphoria revealed a larger decrease in event-related alpha power in response to pleasant stimuli than the group with dysphoria (Fig. 3, panels b,c). It is intriguing to note that at central and centro-parietal scalp sites, the difference between groups in response to pleasant stimuli was lateralized to the right hemisphere, whereas no lateralization was observed at frontal and fronto-central scalp sites (Fig. 3, panel a). It is also worth noting that no significant differences between groups in response to neutral and unpleasant stimuli were noted (Fig. 3, panel b).

#### Differences between groups in event-related delta, theta and beta power for each emotional category

Unpaired t-test conducted on event-related power of other EEG frequency bands averaged over the significant time windows (delta: 100–972 ms; theta: 186–724 ms; beta: 750–1064 ms), where the effect of emotion emerged in the ANOVAs, did not reveal any significant cluster for the difference between the groups within each emotional condition.

## Discussion

The present study investigated motivational deficits in depression during the passive viewing of emotional pictures, according to the capability model of alpha asymmetries. A time-frequency approach was used to examine event-related changes in alpha power in individuals with dysphoria vs. healthy controls with high temporal resolution. Based on previous literature reporting reduced approach-related motivation in depression^10–12,33,35,37^, individuals with dysphoria were expected to show less alpha desynchronization in the left frontal and the right posterior regions in response to pleasant pictures compared to controls.

In line with our hypothesis, individuals with dysphoria showed less alpha desynchronization in response to pleasant stimuli than the group without dysphoria. The effect was evident between 538–1400 ms post-stimulus and was seen at frontal, fronto-central, central and centro-parietal scalp sites. It is important to note that event-related changes in alpha power in response to unpleasant and neutral stimuli were comparable between the two groups. In addition, no difference between groups emerged for delta, theta and beta power, indicating that the present findings were specific for alpha power. Accordingly, these results suggest that depression is characterized by reduced activation of the appetitive (approach) motivational system^8,10,11^.

However, only partially in line with our hypothesis, the difference between groups in response to pleasant stimuli was lateralized to the right hemisphere at centro-parietal scalp sites, whereas no evident lateralization was observed at frontal and fronto-central scalp sites. The present result reflects a reduced cortical activation over bilateral anterior and right-lateralized centro-parietal regions during the processing of pleasant stimuli in individuals with dysphoria as compared to controls. In turn, this suggests that a decreased cortical activation in a network involving bilateral frontal and right-lateralized parietal regions may provide a specific measure of deficits in the Approach Motivation construct within the Positive Valence Systems proposed by the NIMH Research Domain Criteria (RDoC)^52^.

Along the same line of reasoning, the present results provide support for the capability model of individual differences in alpha asymmetry at posterior, but not at anterior scalp sites. It can be suggested that lateralized event-related alpha power at central and centro-parietal scalp sites is more likely to reflect depression-related deficits in the processing of motivationally relevant stimuli than frontal alpha asymmetry. However, the latter result is at odds with findings of previous studies reporting that frontal alpha asymmetry discriminates individuals with depression from healthy controls in approach-related and withdrawal-related conditions^29,30^. An explanation for the discrepant findings may lie in the fact that changes in event-related alpha power were examined in the millisecond range, whereas other studies typically computed alpha activity (and its asymmetries) over longer periods of time^29^. It is worth noting that the effect reported here was robust because the cluster-based analysis allows to correct for multiple comparisons across electrodes and time points.

In the whole group, the present study showed greater alpha desynchronization in response to high-arousing emotional (pleasant and unpleasant) compared to low-arousing neutral stimuli in the 538–1400 ms time window, suggesting that decrease in event-related alpha power may reflect arousal dimension. This interpretation is consistent with recent findings showing a greater alpha desynchronization in response to high-arousing (i.e., erotic) rather than low-arousing (i.e., romantic) pleasant pictures between 600–1000 ms post-stimulus at anterior and posterior scalp sites^53^. Similarly, a decrease in event-related alpha power has been reported to be associated with higher arousal for both pleasant and unpleasant stimuli, with the largest alpha desynchronization occurring in response to erotic and mutilation pictures^54^. It has to be noted that results regarding changes in event-related alpha power in response to emotional stimuli have also reported null findings^55^ or opposite effects (alpha synchronization^56,57^). However, these studies varied remarkably in terms of critical methodological aspects such as central or lateralized presentation of the stimuli, data analysis technique to calculate alpha oscillations, limited number of sensors and picture exposure duration.

In addition, in the whole group ERP results showed the presence of the P3/LPP complex, with larger amplitude occurring in response to pleasant and unpleasant than neutral stimuli at centro-parietal and parietal scalp sites in the 400–604 ms time window. Replicating P3/LPP modulations to high-arousing emotional stimuli compared to low-arousing neutral stimuli with predicted polarity, topography and latency confirmed the effectiveness of the experimental manipulation. It is well-established that P3/LPP complex reflects continued allocation of attention to emotional stimuli and facilitated processing and encoding of motivationally relevant stimuli (for a review, see Lang & Bradley^40^). Our data are in line with those of previous studies showing that LPP and alpha desynchronization may reflect similar processes^54^. However, it is worth noting that the group with dysphoria and the group without did not differ in P3/LPP amplitude in none of the three emotional conditions. In turn, our data suggest that alpha desynchronization over specific brain regions and at specific latencies may represent a more sensitive measure of depression-related motivational deficits than P3/LPP complex. This suggestion is consistent with the notion that event-related oscillations not only reflect stimulus-evoked oscillations similar to the ERPs but also induced oscillations, which are not phase-locked to the stimulus event. It is therefore possible that event-related oscillations may carry important information about emotional processing, which is not represented in the ERPs^58^. Further studies are needed to test differences in emotional processing reflected by ERP and time-frequency analyses.

At the subjective level, self-report measures of valence and arousal did not differ between the group with dysphoria and the control group, in line with previous studies in participants with subclinical depressive symptoms^59,60^ or in patients with clinically significant depression^61,62^ (but see also Sloan *et al*.^63^). An explanation for this null finding is that emotional experience was assessed according to a dimensional model of affective space instead of a discrete emotion model, which may best capture depression-related differences at the subjective level (see Rottenberg *et al*.^3^). Otherwise, it can be suggested that decreased bilateral frontal and right-sided posterior activation may precede alterations in subjective reports of emotional experience and therefore provide an early measure of deficits in the appetitive motivational system in individuals with dysphoria.

With respect to clinical implications, the current data show that individuals with dysphoria are characterized by under-engagement of appetitive rather than over-engagement of aversive motivational system^7,10,64^. In line with this finding, there is recent evidence showing that depressed mood may improve through interventions specifically aimed at increasing appetitive motivation^65^. It can be also suggested that underactivation of the approach-related motivational system in at-risk individuals (e.g., with dysphoria) may be involved as a risk factor for the development of a full-blown depressive episode. Consistent with this suggestion, the clinical manifestation and the course of depression have been reported to be worsened by underactivation of the appetitive motivational system^66^. However, longitudinal studies are needed to test whether decreased approach-related motivational drive may play a role in the transition from dysphoria to major depression.

The present study suffers from some methodological shortcomings. First, the sample size included in this study was relatively small and, second, it was composed almost exclusively by females. Therefore, the current findings need to be replicated and extended to males in order to increase their generalizability. Third, whether participants included in the study met the criteria for major depression, dysthymia or bipolar disorder in the past was not investigated. Although having a history of major depression is unlikely to have affected the results obtained in the group with dysphoria because the effect of current and past depression on alpha asymmetry is expected to be the same, it might have partially confounded data obtained in healthy controls. It should be noted, however, that 12-month period prevalence of depression range from 1% to 3% in pre-pubertal children and post-pubertal adolescents^67^. According to this prevalence rate, and given that only university students were enrolled, the likelihood of having a history of clinically significant depression in participants assigned to healthy control group was very low. Therefore, the potential confounding effect of having a history of major depression was limited in the present study. Lastly, the module A of the SCID-I interview was administered by only one trained psychologist, which prevented us from evaluating the inter-rater reliability. Nonetheless, a high inter-rater reliability has been previously reported for the majority of the disorders assessed by the SCID-I interview^68^.

To the best of our knowledge, this is the first study investigating motivational deficits in depression using a time-frequency approach, according to the capability model of alpha asymmetries. The excellent temporal resolution of this approach gave us the opportunity to use discrete, short-lasting emotional stimuli needed to elicit a strong activation of the approach- and withdrawal-related motivational systems, as proposed by the capability model. In other words, the time-frequency approach allowed us to go beyond the measurement of a trait-like deficit in approach motivation, detailing how depressed mood affects transient motivational responses.

In conclusion, the results obtained in the present study support the notion that individuals with dysphoria are more likely to be under-engaged in processing approach- than avoidance-related motivationally stimuli rather than the opposite pattern. Most importantly, these novel results add to the existing literature by suggesting that transient reduction in alpha desynchronization involving bilateral frontal and right-lateralized parietal regions may reflect deficits in the approach-related motivational system in depression.

## Data Availability

All data and MATLAB code will be made available upon request.
